# Supplementary figures and images for: Effects of water decontamination methods and bedding material on the gut microbiota
Source: PLoS One. 2018 Oct 25;13(10):e0198305. doi: 10.1371/journal.pone.0198305 (PMC6201873; doi:10.1371/journal.pone.0198305)

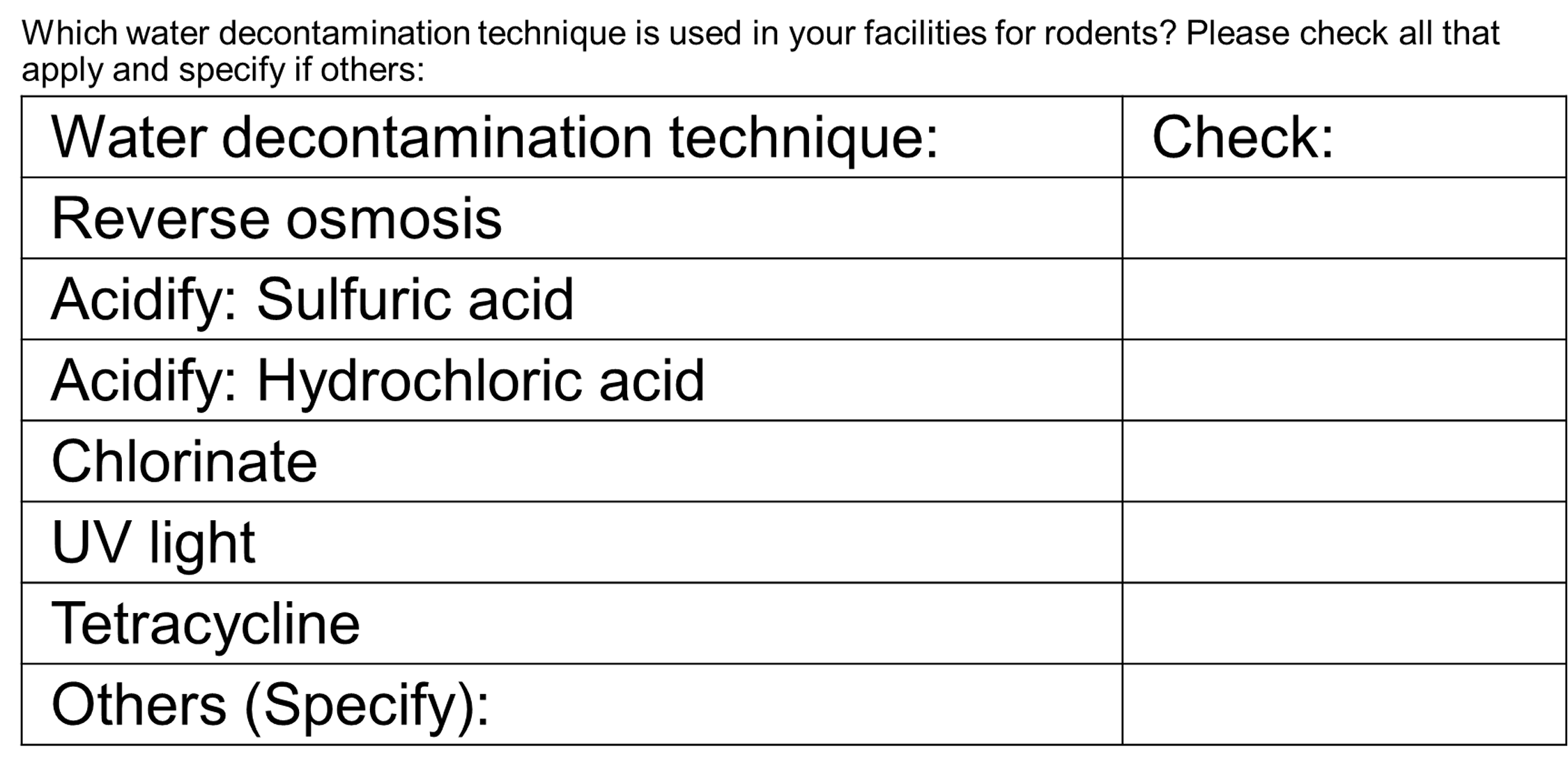

Supplement: S1 Table — Survey sent via e-mail through Compmed listserv on December 15, 2015. (TIF) [file pone.0198305.s001.tif]

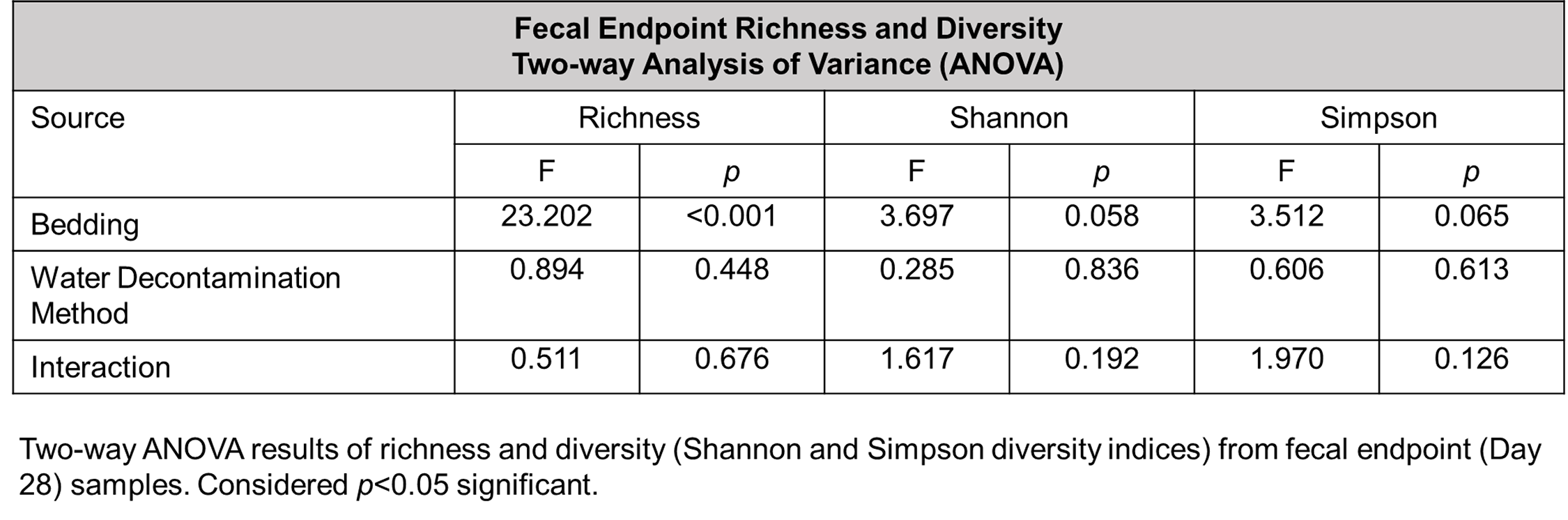

Supplement: S2 Table — Two-way ANOVA results of richness and diversity (Shannon and Simpson diversity indices) from fecal endpoint (Day 28) samples. Considered p<0.05 significant. (TIF) [file pone.0198305.s002.tif]

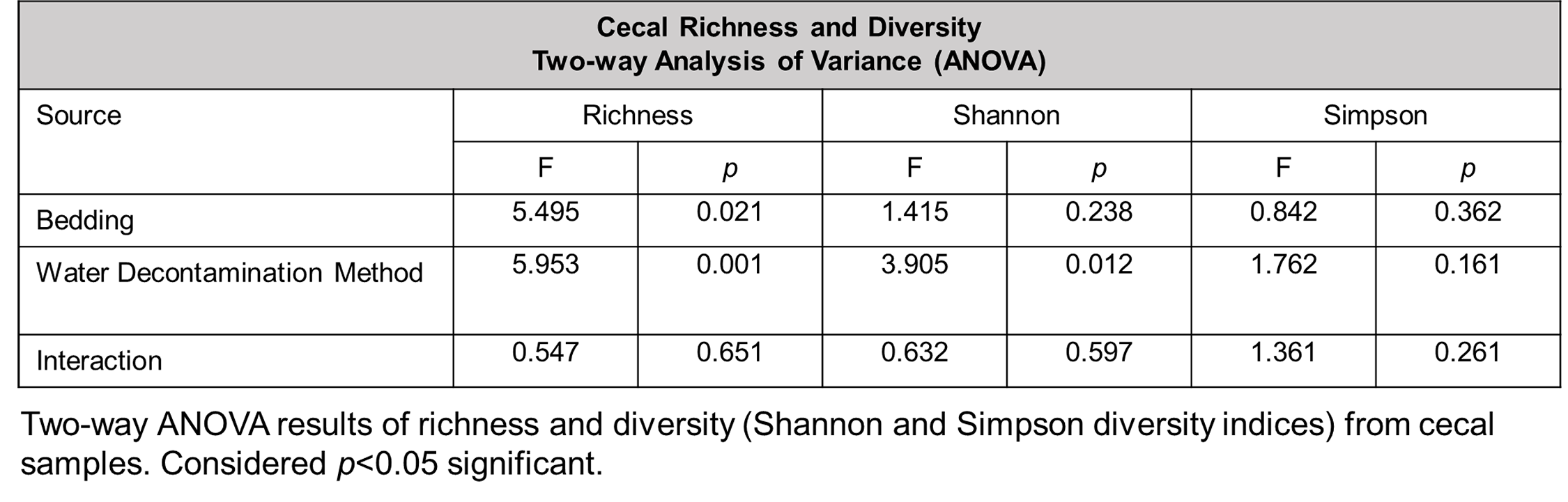

Supplement: S3 Table — Two-way ANOVA results of richness and diversity (Shannon and Simpson diversity indices) from cecal samples. Considered p<0.05 significant. (TIF) [file pone.0198305.s003.tif]

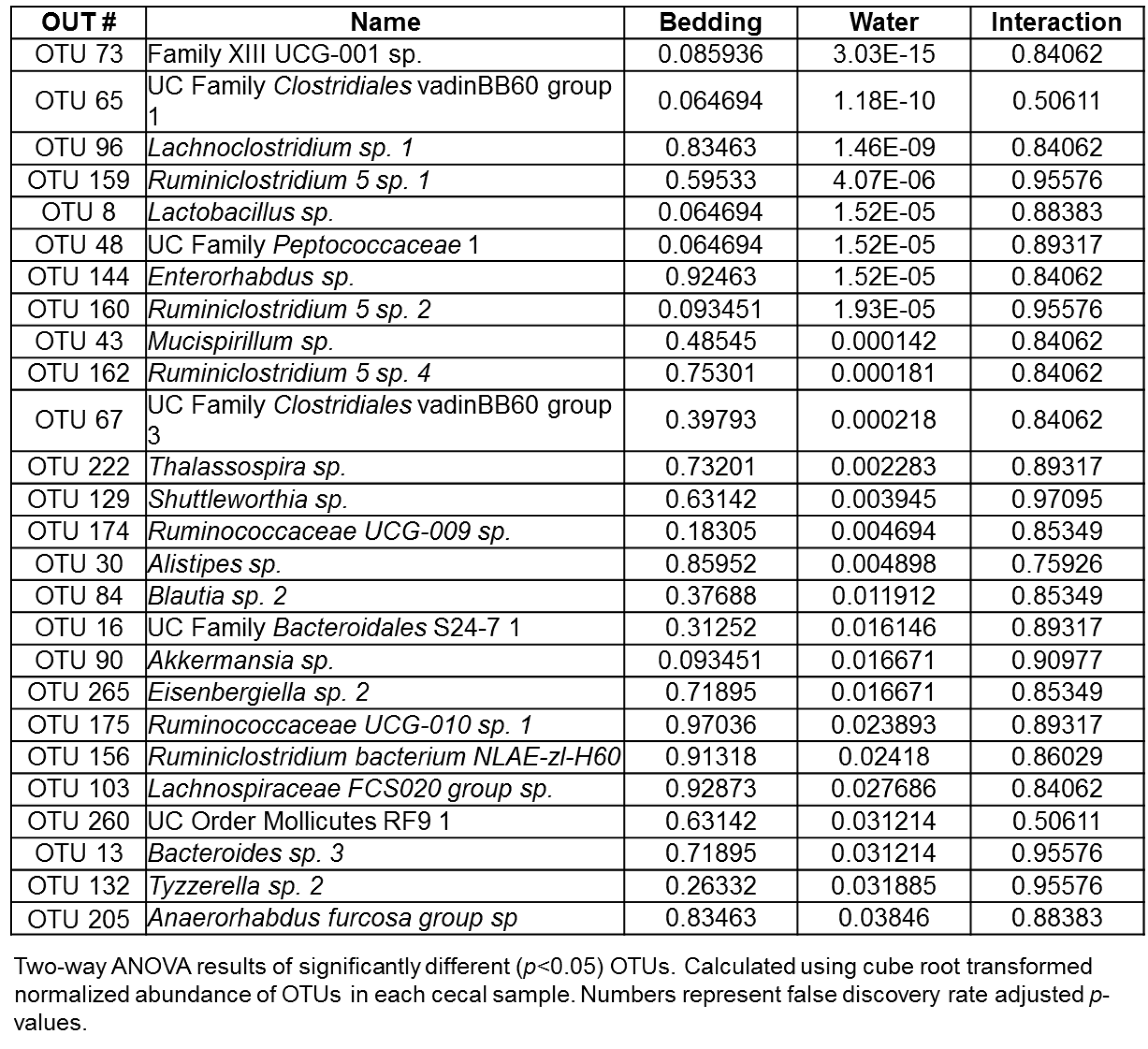

Supplement: S4 Table — Two-way ANOVA results of significantly different (p<0.05) OTUs. Calculated using cube root transformed normalized abundance of OTUs in each sample. Numbers represent false discovery rate adjusted p-values. (TIF) [file pone.0198305.s004.tif]

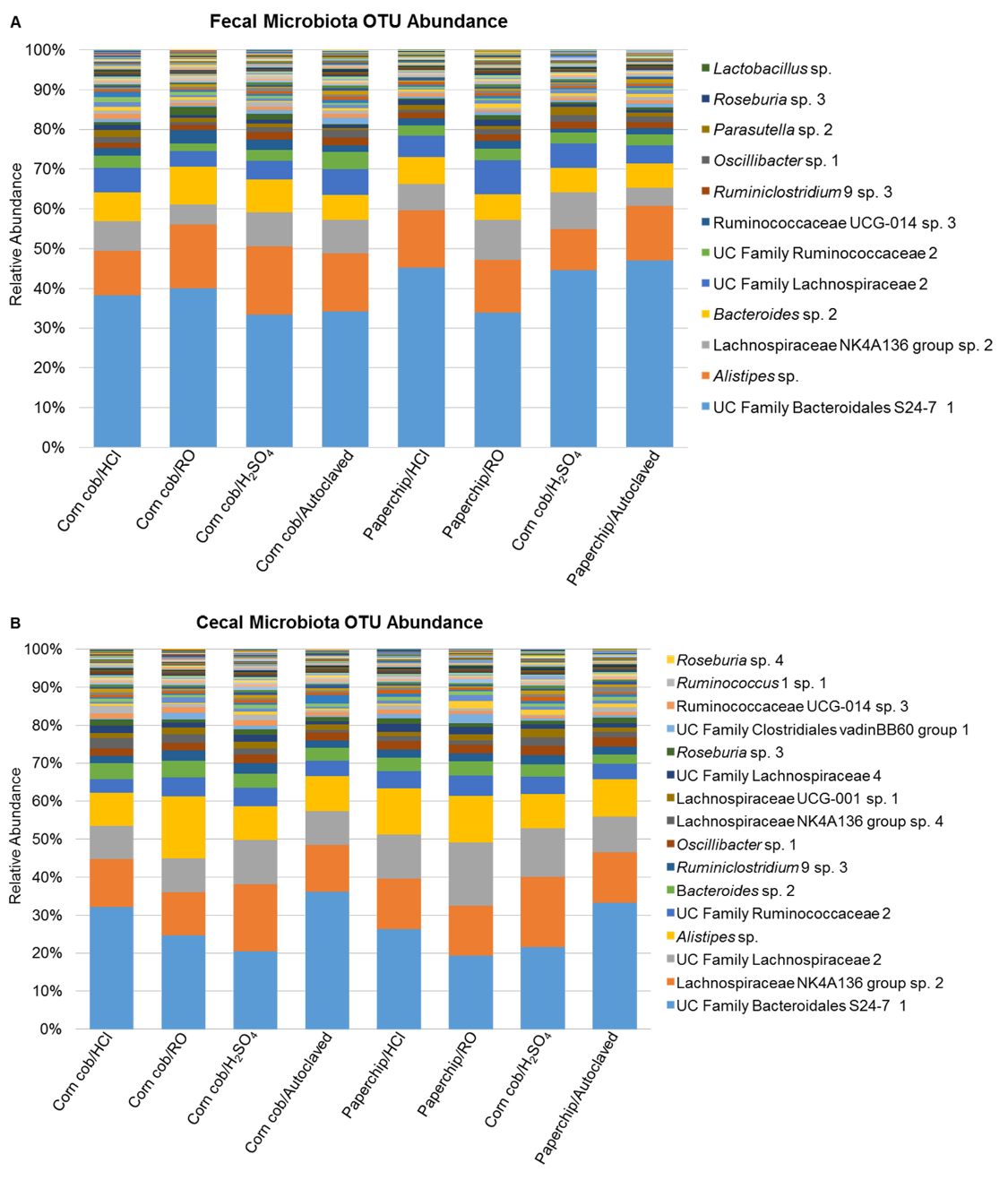

Supplement: S1 Fig — Bar graphs representing the average relative abundance of OTUs in each group for (A) fecal microbiota and (B) cecal microbiota. Each color represents a different OTU. Legend on right represents OTUs with high (>1%) relative abundance. (TIF) [file pone.0198305.s005.tif]

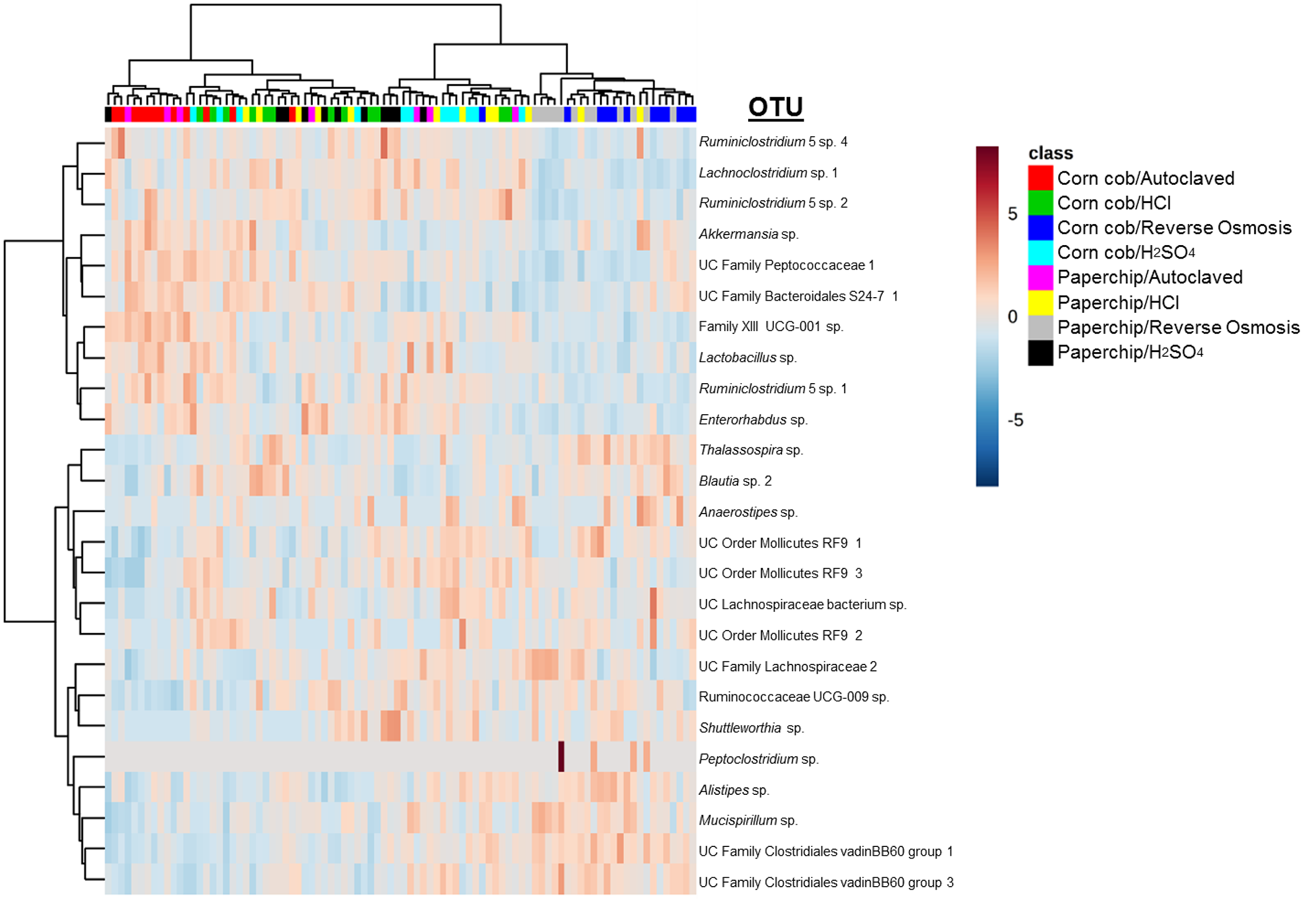

Supplement: S2 Fig — Hierarchical clustering of the top 25 (lowest p-values corrected by false discovery rate) OTUs by one-way ANOVA of all cecal samples. Color intensity shows cube root transformed normalized abundance of OTUs in each sample. Color-coded bars at top represent bedding/water group (legend on right). (TIF) [file pone.0198305.s006.tif]

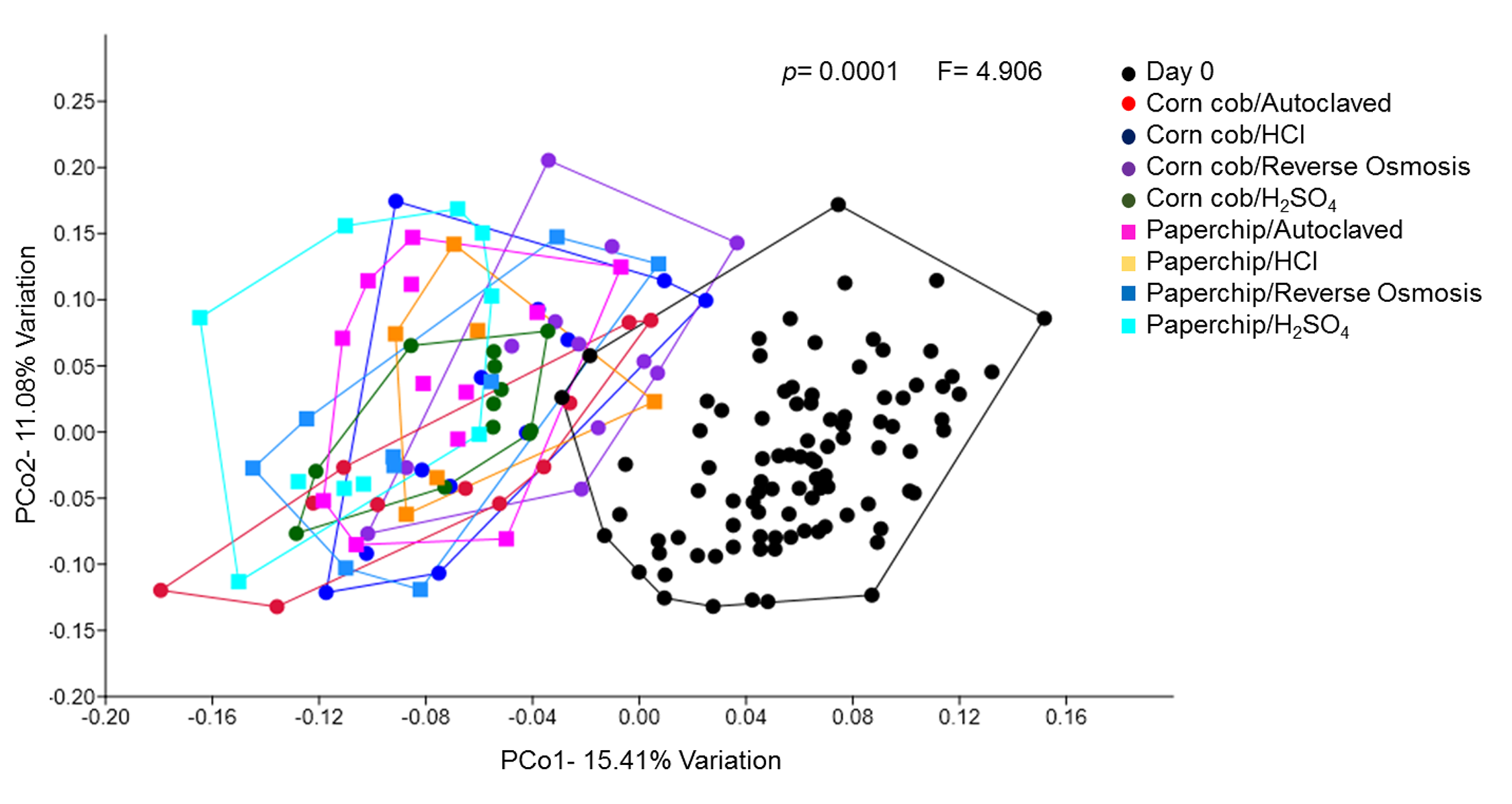

Supplement: S3 Fig — Principal coordinate analysis of all fecal samples at arrival (black circles) and all different endpoint groups (legend on right). One-way PERMANOVA of ranked Bray-Curtis similarity indices results shown. (TIF) [file pone.0198305.s007.tif]
